# Correction: Consistent movement of viewers’ facial keypoints while watching emotionally evocative videos

**DOI:** 10.1371/journal.pone.0321320

**Published:** 2025-03-27

**Authors:** The PLOS One Staff

The Data Availability statement for this paper is incorrect. The correct statement is: The DISFA dataset used in the current study is publicly available at the link http://mohammadmahoor.com/disfa/ by filling out an agreement form. All the Python code used in the analysis, including those for generating preprocessed data and the statistical models, is available at https://github.com/Shivansh-ct/Consistent-Keypoints.

The publisher apologizes for the error.
